# The Chemists' Exhibition

**Published:** 1902-06-21

**Authors:** 


### THE CHEMISTS' EXHIBITION.

This annual exhibition was held at St. James's Hall, Manchester, from June 3rd to June 6th, and attracted a considerable attendance of both medical men and nurses. The extensive show of drugs, pharmaceutical requisites, and sundries included many new preparations and appliances. Messrs. Evans, Sons, and Co. had a most effective display, in which were specimens of their crepe and selvedge bandages, thyro-glandin and alginoid iron (Stanford) while Messrs. Parke, Davis, and Co. gave an excellent exhibit, including euthymol preparations, and cascara evacuant, which they describe as a tasteless preparation of the drug. Messrs. Wyleys, Limited, showed their medicated pastilles, malt preparations, and malted food, and Messrs. Oppenheimer their palatinoids and bi-palatinoids, also renaglandin, an extract of suprarenal gland containing ephederin and superinine. The stall of Messrs. Woolley, Sons, and Co., Limited, was of much interest, their Decalcified Cancellous Tissue and soluble medicated ear cones being specially observed, whilst syrup hypophosph. comp., and liq. anemone co., a sedative, were exhibited by Messrs. A. Newton and Co. Messrs. Shirley Bros., Limited, had a new feeding bottle made on the latest hygienic principles. Food preparations were exhibited by Messrs. Mellins, Limited, with their well-known food and biscuits; Cheltine Foods, Limited, with their diabetic white and brown bread, flour, and milk cocoa (containing 50 per cent. cheltine powdered milk); whilst the Maltova Food Company and Messrs. Strenbo, Limited, exhibited their preparations.

The Vinolia Company, Limited, gave a beautiful display of their high-class soaps and toilet preparations, and the Erasmic Company, Limited, also showed their soaps and toilet requisites to much advantage. The Holme Preparations Company exhibited their Malloween and Malloween toilet powder, and the Crown Perfumery Company their carbolic and coal tar soaps for toilet use. With regard to mineral waters, Vichy, Hunyadi Janos, and Carlsbad Water were shown by Messrs. Ingram and Royle, while Rosbach was much in evidence. Camwal, Limited, exhibited aërated and medicated waters; also an apparatus for testing the purity of the waters.

There was a most interesting display of bandages and dressings, including porous belladonna plaster and the "Leicester" breast plaster, by Messrs. A. De St. Dalmas and Co.; while Messrs. Wm. Mather, Limited, with surgeons' rubber adhesive plasters and belladonna plasters of guaranteed strength, claimed much attention.

